# Cytosolic Hsp90α and its mitochondrial isoform Trap1 are differentially required in a breast cancer model

**DOI:** 10.18632/oncotarget.15659

**Published:** 2017-02-23

**Authors:** Evangelia Vartholomaiou, Marta Madon-Simon, Stéphane Hagmann, Guillaume Mühlebach, Wolfgang Wurst, Thomas Floss, Didier Picard

**Affiliations:** ^1^ Département de Biologie Cellulaire, Université de Genève, Sciences III, Genève, Switzerland; ^2^ Helmholtz Zentrum München, Deutsches Forschungszentrum für Gesundheit und Umwelt, Neuherberg, Germany; ^3^ Deutsches Zentrum für Neurodegenerative Erkrankungen e. V., München, Germany; ^4^ Munich Cluster for Systems Neurology, München, Germany; ^5^ Technische Universität München-Weihenstephan, Neuherberg, Germany

**Keywords:** Hsp90, Trap1, breast cancer, metastasis, mouse model

## Abstract

The Hsp90 family of molecular chaperones includes the cytosolic isoforms Hsp90a and Hsp90β and the mitochondrial isoform Trap1. Hsp90a/βsupport a large number of client proteins in the cytoplasm and the nucleus whereas Trap1 regulates oxidative phosphorylation in mitochondria. Many of the associated proteins and cellular processes are relevant to cancer, and there is ample pharmacological and genetic evidence to support the idea that Hsp90a/βand Trap1 are required for tumorigenesis. However, a direct and comparative genetic test in a mouse cancer model has not been done. Here we report the effects of deleting the *Hsp90a* or *Trap1* genes in a mouse model of breast cancer. Neither Hsp90a nor Trap1 are absolutely required for mammary tumor initiation, growth and metastasis induced by the polyoma middle T-antigen as oncogene. However, they do modulate growth and lung metastasis *in vivo* and cell proliferation, migration and invasion of isolated primary carcinoma cells *in vitro*. Without Hsp90a, tumor burden and metastasis are reduced, correlating with impaired proliferation, migration and invasion of cells in culture. Without Trap1, the appearance of tumors is initially delayed, and isolated cells are affected similarly to those without Hsp90a. Analysis of expression data of human breast cancers supports the conclusion that this is a valid mouse model highlighting the importance of these molecular chaperones.

## INTRODUCTION

Members of the Heat shock protein 90 (Hsp90) family of molecular chaperones are highly conserved, ubiquitous and abundant ATP-dependent proteins with different isoforms that share a high degree of sequence identity and can be found in several cellular compartments: Hsp90α and Hsp90β in the cytoplasm and the nucleus, here referred to generically as Hsp90, Trap1 in mitochondria, Grp94 in the endoplasmic reticulum, and Hsp90C in chloroplasts [1]. The cytosolic Hsp90 is a key component of an ensemble of complexes with a variety of co-chaperones [2], which acts on a wide range of protein substrates called clients (for a comprehensive overview, see https://www.picard.ch/downloads/Hsp90interactors.pdf and ref. 3).

It has been demonstrated that cytosolic Hsp90 is overexpressed in several types of cancers [3]. Moreover, Hsp90 was found to interact with a number of proteins important for breast cancer, for example the hypoxia inducible factor HIF-1α, estrogen receptor α, anti-apoptotic kinase Akt, tumor suppressor protein p53, and the ErbB receptor tyrosine kinase (see https://www.picard.ch/downloads/Hsp90facts.pdf for a comprehensive overview and references). The Hsp90 chaperone machinery can be important for tumorigenic transformation due to its ability to stabilize overexpressed or mutated oncoproteins or transformation-relevant signaling pathways, thereby contributing to oncogene addiction and survival of cancer cells [4–6]. Hsp90α has been proposed to promote the motility, invasiveness and acquisition of stress resistance of cancer cells [7–9]. Importantly, some studies have shown that the overexpression of Hsp90β in breast cancer, as part of a set of proteins involved in regulating estrogen receptor α activity, correlates with adverse clinical outcomes [10]; a variety of Hsp90 inhibitors are in clinical trials for breast cancer treatment [11], further highlighting the potential of Hsp90 as a therapeutic target.

In addition to the intracellular pool of Hsp90, the discovery of extracellular Hsp90α (eHsp90α) secreted by several normal and tumor cell lines has made the understanding of Hsp90α biology even more challenging [7, 12]. eHsp90α has been proposed to be important in a number of physiological and pathophysiological processes including tumor growth [13], invasion and metastasis [7, 14], wound healing [15], and angiogenesis [16]. It has been suggested that Hsp90 secretion in normal cells only occurs under stress, but is constitutive in certain tumor cells [17], although not in all [18]. Thus, the selective sensitivity of tumor cells to Hsp90 inhibitors could be due to the inhibition of eHsp90 rather than or in addition to that of intracellular Hsp90 [17]. Since migration, invasion and metastasis are not necessarily dependent on eHsp90 in all types of cancer cells, it was speculated that sensitivity to Hsp90 inhibitors may be restricted to a subgroup of eHsp90-dependent cells [12, 17]

Initial evidence for the functions of the mitochondrial Hsp90 isoform Trap1 [19] indicated a significant protective role of this molecular chaperone against oxidative stress and cell death [20]. More recent studies have highlighted the importance of Trap1 in mitochondrial physiology and notably in the regulation of the balance between mitochondrial respiration and aerobic glycolysis [21]. However, there are contradictory conclusions among different studies as to whether Trap1 inhibits or stimulates mitochondrial respiration, indicating a complex and maybe contextual role. Since tumors rewire their metabolism towards the glycolytic pathway, even in the presence of oxygen, a phenomenon referred to as the Warburg effect [22], changes in Trap1 protein levels might facilitate this metabolic switch [21, 23]. Alterations of Trap1 expression have been reported in different types of cancer. It is overexpressed in human ovarian carcinomas resistant to platinum drugs [24], in multi-drug resistant human colorectal carcinomas [25], and in prostate [26, 27], pancreatic and lung cancers [27]. In contrast, in renal cell carcinoma, bladder and cervical cancers, Trap1 protein levels are lower when compared to normal tissue and they display an inverse correlation with tumor stage [23]. Additionally, in ovarian cancer lower levels of Trap1 were associated with more advanced disease [28]. For breast cancer, increased expression of Trap1 has been reported [27, 29]. Using a breast cancer xenograft model, a recent study showed decreased tumorigenesis when Trap1 expression was knocked down, whereas overexpression of Trap1 resulted in decreased metastasis [29].

Due to the paucity of genuine *in vivo* studies on the roles of Hsp90α and Trap1 in breast cancer and given the complexity of the above-mentioned observations, we decided to investigate the role of these two Hsp90 isoforms for breast cancer initiation, progression and metastasis genetically in a mouse model. Genetically engineered mouse cancer models possess several advantages over xenograft models: immunocompetent mice can be used, authentic tumor-stroma interactions are maintained, and the process of metastasis from the primary tumor may be recapitulated [30, 31]. For these reasons, we took advantage of a mouse strain carrying the oncogene polyoma virus middle T-antigen (PyMT) under the control of the mouse mammary tumor virus long terminal repeat [32]. The expression of the PyMT transgene results in the rapid development of breast adenocarcinomas with a high incidence of pulmonary metastasis [32], and it has been shown to be an adequate model to mimic human invasive ductal carcinoma [33]. Given that Hsp90-null [34] and Trap1-null [35, 36] mice are viable, we decided to investigate the importance of Hsp90α and Trap1 for mammary tumorigenesis by introducing the PyMT oncogene into Hsp90α- and Trap1-null mice. These genetic experiments in the mouse unambiguously address the importance of these molecular chaperones, at least for this particular model of breast cancer, and allow us to speculate about their relevance to human breast cancer.

## RESULTS

### Expression of Hsp90α and Trap1 in breast tumors and metastatic nodules

**Figure 1:**
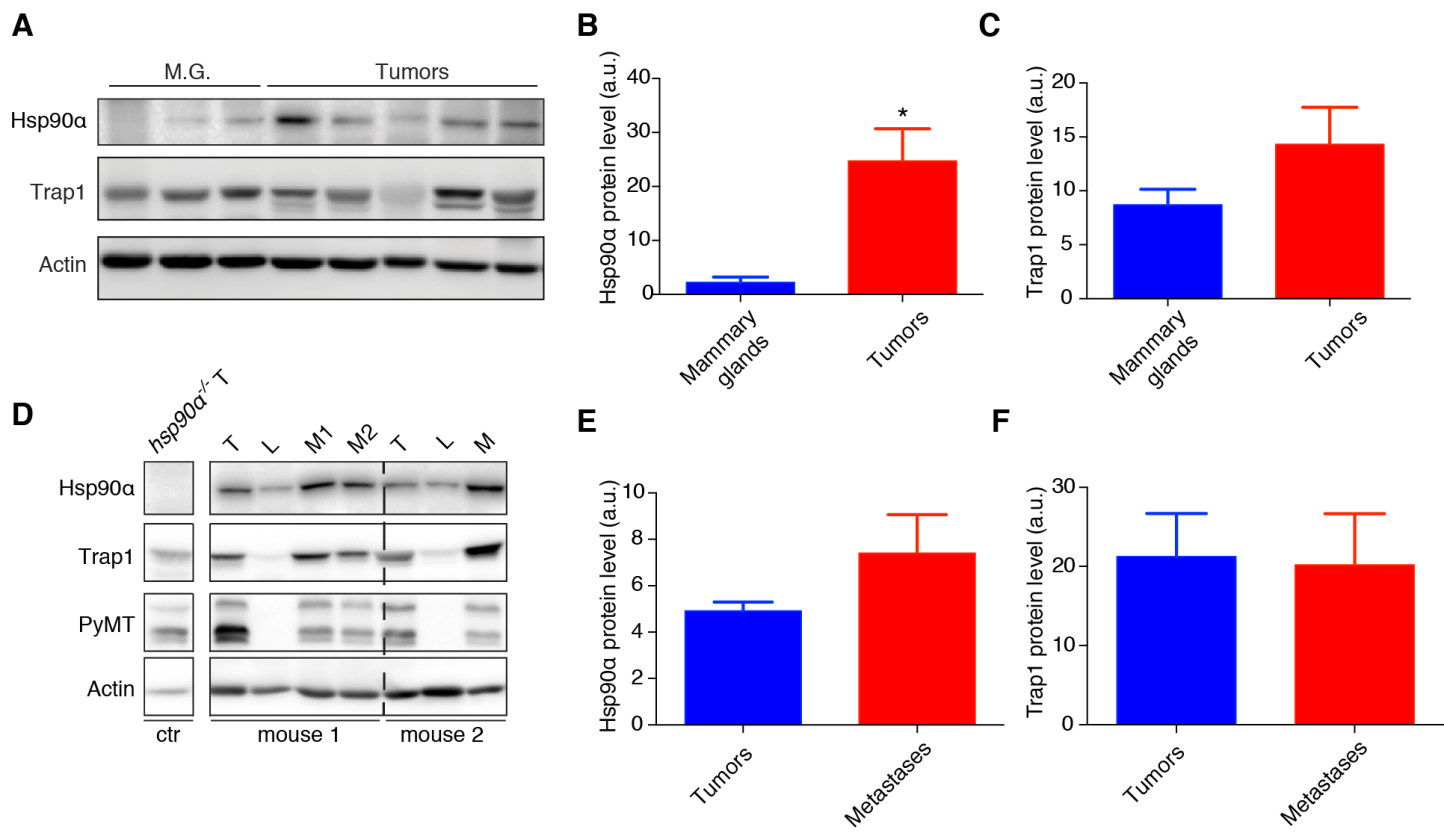
Expression levels of Hsp90α and Trap1 in mammary tumors and metastatic nodules. **A**. Immunoblot showing Hsp90α and Trap1 protein levels in mammary glands (M.G) derived from mice without the PyMT transgene (*n* = 3) and tumors from mice expressing the PyMT transgene (*n* = 5); actin was used as loading control. **B**. and **C**. Bar graphs with the quantitation of the immunoblots; a significant increase of Hsp90α level in tumors is indicated by an asterisk (*p < 0.05); note that the apparent increase of Trap1 in tumors is not statistically significant. **D**. Immunoblot showing protein levels of Hsp90α and Trap1 in tumors (T), metastatic nodules (M) and normal lung tissue (L) from two mice with the PyMT transgene; proper dissection of normal lung tissue was confirmed by the absence of PyMT; actin was used as loading control. **E**. and **F**. Quantitation of the immunoblots; comparing metastatic nodules and tumors, Hsp90α levels do not change in a statistically significant manner (*n* = 6 mice) nor do Trap1 levels (*n* = 5).

To obtain initial correlative evidence for the potential role of Hsp90α and Trap1 in the tumorigenic and metastatic processes in the PyMT breast cancer model, we checked their protein levels in normal and cancer tissues. Hsp90α levels are significantly increased in tumors compared to normal mammary gland tissue (Figure 1A and 1B), whereas Trap1 expression levels do not significantly change (Figure 1A and 1C). Our next question was whether there was any change in the protein levels of Hsp90α and Trap1 in metastases compared to the primary tumors and to normal lung (Figure 1D and Supplementary Figure 1A and B). We observed a slight but not statistically significant increase in Hsp90α levels in metastases compared to primary tumors (Figure 1E) and no change in Trap1 levels (Figure 1F). Significantly higher protein levels of both Hsp90α (Supplementary Figure 1C) and Trap1 (Supplementary Figure 1D) were noted in metastatic nodules when compared to normal adjacent lung tissue. Thus, the presence of Hsp90α and Trap1 through all stages of tumorigenesis is compatible with their involvement in these processes.

### Effects of deleting the *Hsp90α* and *Trap1* genes on tumor initiation and progression

**Figure 2:**
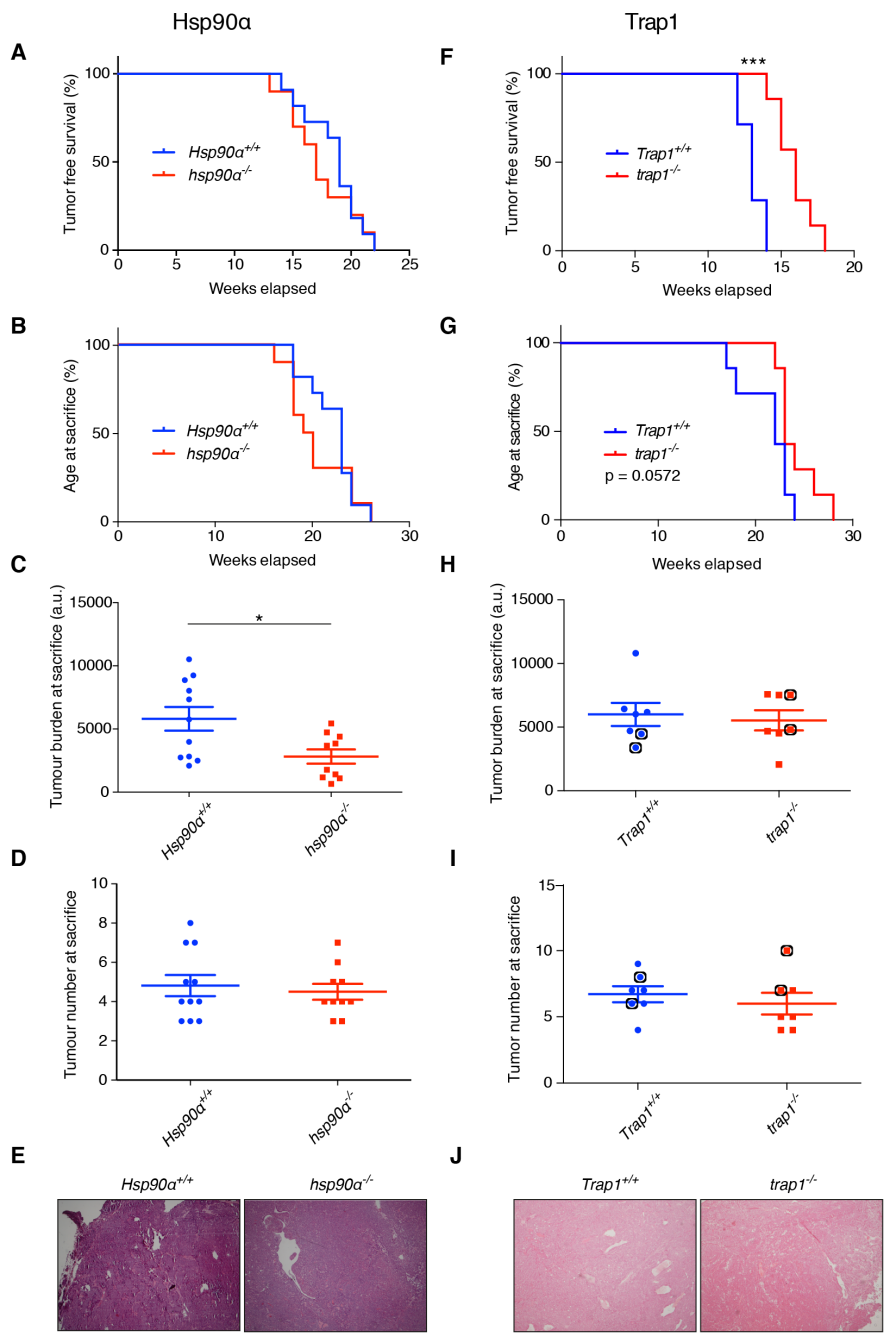
Effects of deleting the ***Hsp90α*** and ***Trap1*** genes on tumor onset and growth. **A**. to **E**. Comparisons between *Hsp90α*+/+ and *hsp90α*−/− mice. Kaplan-Meier curves showing no difference in (**A**.) tumor free survival and (**B**.) age at sacrifice. **C**. Significant decrease in tumor burden in *hsp90α*−/− mice at sacrifice (*p < 0.05). **D**. No difference in tumor numbers at sacrifice. **E**. Tumor histology assessed by H&E indicating no difference (magnification x40, representative images are shown). **F**. to **J**. Comparisons between *Trap1*+/+ and *trap1*−/− mice. **F**. Significantly longer tumor-free survival of *trap1*−/− mice (***p < 0.001); note that the seemingly more rapid tumor development in *Trap1*+/+ compared to *Hsp90α*+/+ (shown in panel a) mice is irrelevant because the results are from totally independent experiments with separate and non-comparable wild-type littermates. **G**. *trap1*−/− mice showed a trend towards longer overall survival (*p* = 0.0572). **H**. Similar tumor burden at sacrifice. **I**. No difference in tumor numbers at sacrifice. Indicated with black circles are the mice from which primary cells were isolated (see Figure 4). **J**. Tumor histology indicating no difference (magnification x40, representative images are shown).

Hsp90α is encoded by the gene *Hsp90aa1*; for clarity, we will refer to it here as *Hsp90α*. To study the effects of its deletion on mammary tumorigenesis, we compared mammary tumor initiation and progression in mice with the PyMT transgene on the two genetic backgrounds (*Hsp90α*+/+ versus *hsp90α*−/−). The first palpable tumor was observed in both groups at a similar age (Figure 2A). We did not observe any significant differences in the overall survival of the two groups (Figure 2B). Interestingly, *hsp90α*−/− mice on average displayed a reduced tumor burden (Figure 2C), but not tumor number (Figure 2D), at sacrifice. The morphology of the tumors was similar in both groups as shown by hematoxylin and eosin (H&E) staining (Figure 2E). This suggests that, in the absence of Hsp90α, there is a defect in tumor growth, but not in tumor initiation.

To examine the effects of the absence of Trap1 on mammary tumorigenesis, we assessed the same parameters by comparing *Trap1*+/+ with *trap1*−/− mice. Interestingly, *trap1*−/− mice developed the first palpable tumors on average 3 weeks later than their wild-type littermates (Figure 2F); this is also reflected in a prolonged overall survival of the *trap1*−/− mice (Figure 2G). However, at sacrifice, tumor burden (Figure 2H) and number (Figure 2I) displayed no differences regardless of the presence or absence of Trap1. The tumor histopathology appeared similar in the two groups (Figure 2J). These observations indicate that loss of Trap1 results in an initial delay in the appearance of the primary tumor, but not in the subsequent tumor growth.

### Absence of Hsp90α, but not of Trap1, impairs the formation of lung metastases

**Figure 3:**
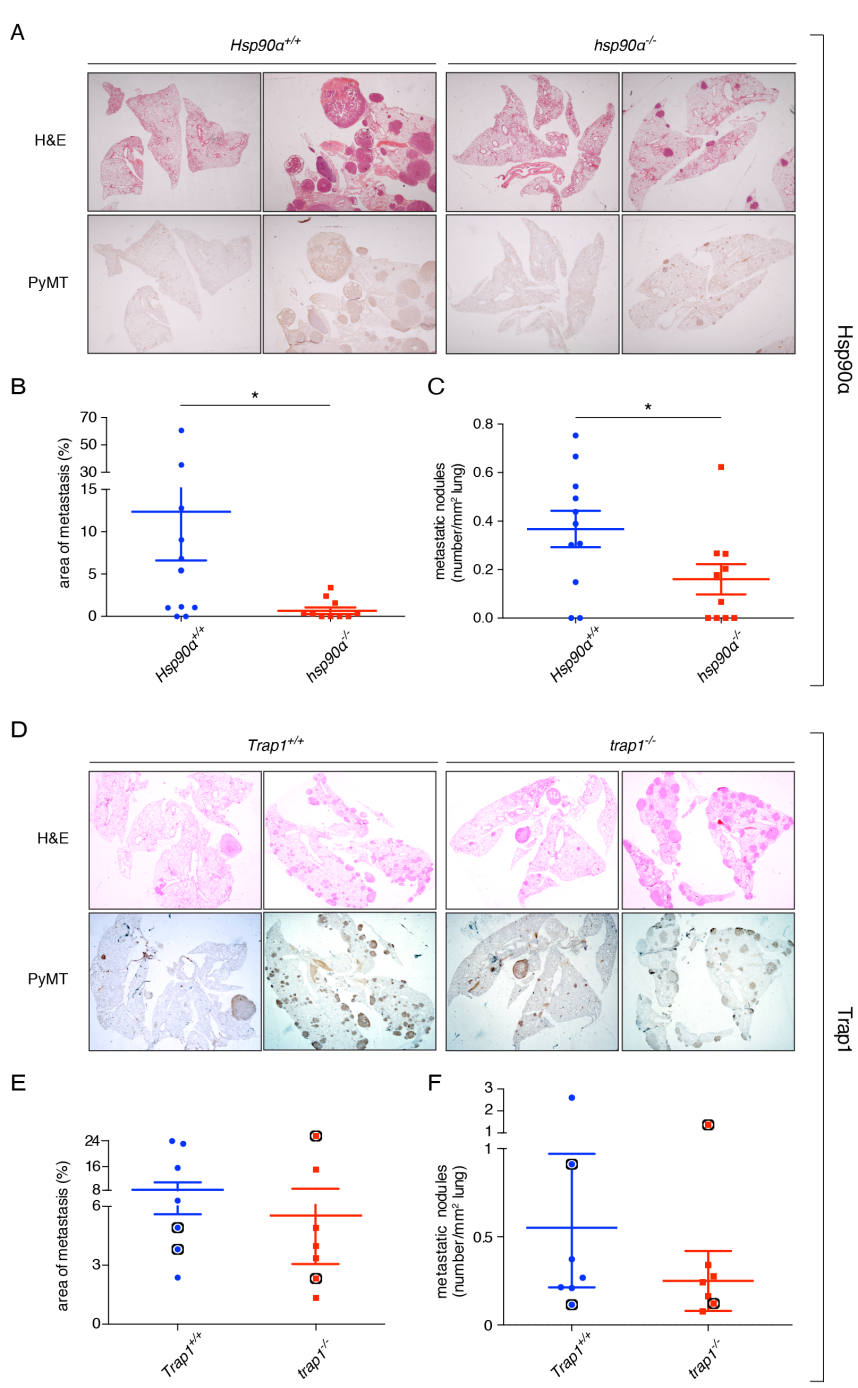
Effects of deleting the ***Hsp90α*** and ***Trap1*** genes on lung metastasis. **A**. and **D**. H&E staining (upper panel) and immunohistochemical staining for the PyMT antigen (lower panel) in whole lung sections of mice with the indicated genotypes (magnification x10, representative images are shown). **B**. Significant decrease in the metastatic area of *hsp90α*−/− lungs (*p < 0.05). **C**. Significant decrease in the total number of metastatic nodules in *hsp90α*−/− lungs (*p < 0.05). **E**. No difference in the area of metastasis between *Trap1*+/+ and *trap1*−/− lungs. **F**. No difference in the number of metastatic nodules between *Trap1*+/+ and *trap1*−/− lungs. Indicated with black circles are the mice from which primary cells were isolated (see Figure 4).

In the PyMT mammary cancer model, lung metastases are observed with high incidence shortly after the appearance of primary tumors [33, 37]. 82% of the wild-type *Hsp90α*+/+ control mice, 60% of the *hsp90α*−/− mice (Supplementary Figure 2A), and all the mice of both Trap1 groups developed lung metastases (Supplementary Figure 2B). Metastatic nodules could be clearly distinguished by H&E staining, and the presence of the PyMT antigen could be detected by immunohistochemistry (Figure 3A and 3D). On average, the loss of Hsp90α resulted in a significant decrease in the total area and number of metastatic nodules (Figure 3B and 3C), even though there were large individual differences. However, no significant differences were observed in the lungs with depletion of Trap1, neither for the area of metastasis nor for the number of the metastatic nodules (Figure 3E and 3F). These results indicate an important role for the cytosolic Hsp90 isoform Hsp90α in the metastatic process.

### Isolated mammary tumor cells display decreased proliferation in the absence of Hsp90α or Trap1

**Figure 4:**
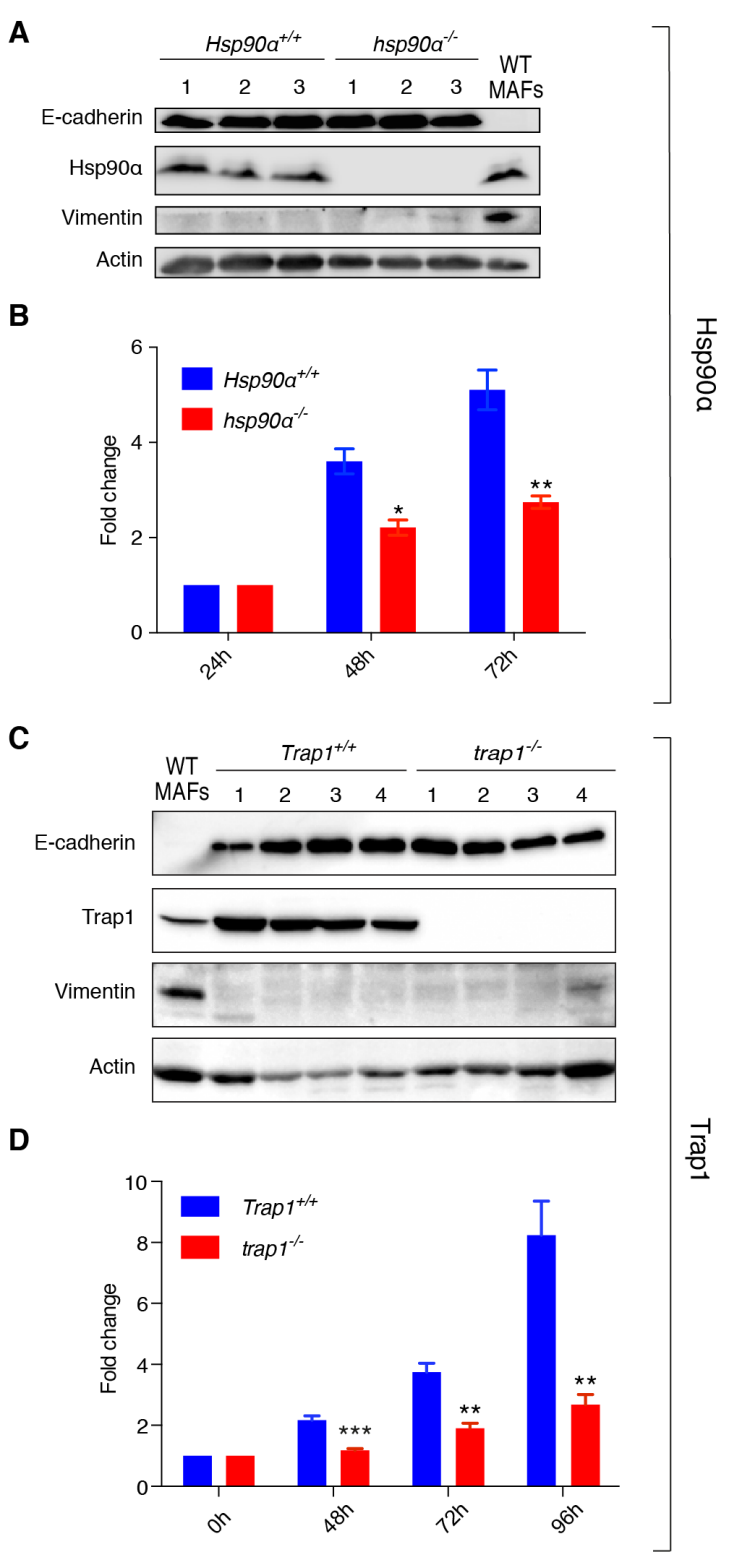
*hsp90α^−/−^* and *trap1^−/−^* cells of epithelial origin derived from mammary tumors show decreased proliferation **A**. and **C**. Immunoblots confirming epithelial origin of isolated cells, showing expression of E-cadherin and lack of expression of vimentin; cells were isolated from 3 *Hsp90α*+/+ and 3 *hsp90α*−/− mice, 2 *Trap1*+/+ and 2 *trap1*−/− animals (2 tumors per mouse); protein extracts from primary mouse adult fibroblasts (MAFs) were used as a positive control for vimentin expression; actin was used as loading control. **B**. *hsp90α*−/− cells exhibited a significant decrease in growth rates (measured by MTT assay) at 48 (*p < 0.05) and 72 hours (**p < 0.01) after seeding. **D**. A decrease in proliferation rates was observed in *trap1*−/− cells (measured by standard cell counting) at 48 (***p < 0.001), 72 (**p < 0.01) and 96 hours (**p < 0.01) post seeding; results of three independent experiments are illustrated as a fold change to the absorbance at 24 hours after seeding (Hsp90α) or to the number of cells seeded (Trap1).

To improve our understanding of the molecular and cellular defects underlying our observations in the mouse model, we decided to characterize primary cells derived from tumors *in vitro*. We confirmed the epithelial origin of the isolated cells by probing immunoblots of cell extracts of all independent cellular isolates for the epithelial marker E-cadherin and for the mesenchymal marker vimentin (Figure 4A and 4C). Due to the lower tumor burden in *hsp90α*−/− mice and the delay in tumor onset in *trap1*−/− animals, we determined the proliferation rates of isolated cells. Interestingly, *hsp90α*−/− cells exhibited significant growth retardation at 48 and 72 hours post seeding (Figure 4B), and similar observations were made for *trap1*−/− cells at 48, 72 and 96 hours post seeding (Figure 4D). Reduced cell numbers were clearly due to reduced proliferation rather than cell death since cells were counted after counterstaining dead ones with trypan blue. These results with isolated primary mammary tumor cells are consistent with an important role of Hsp90α for mammary tumor growth and Trap1 in mammary tumor onset.

### Mouse mammary tumor cells do not secrete Hsp90α

**Figure 5:**
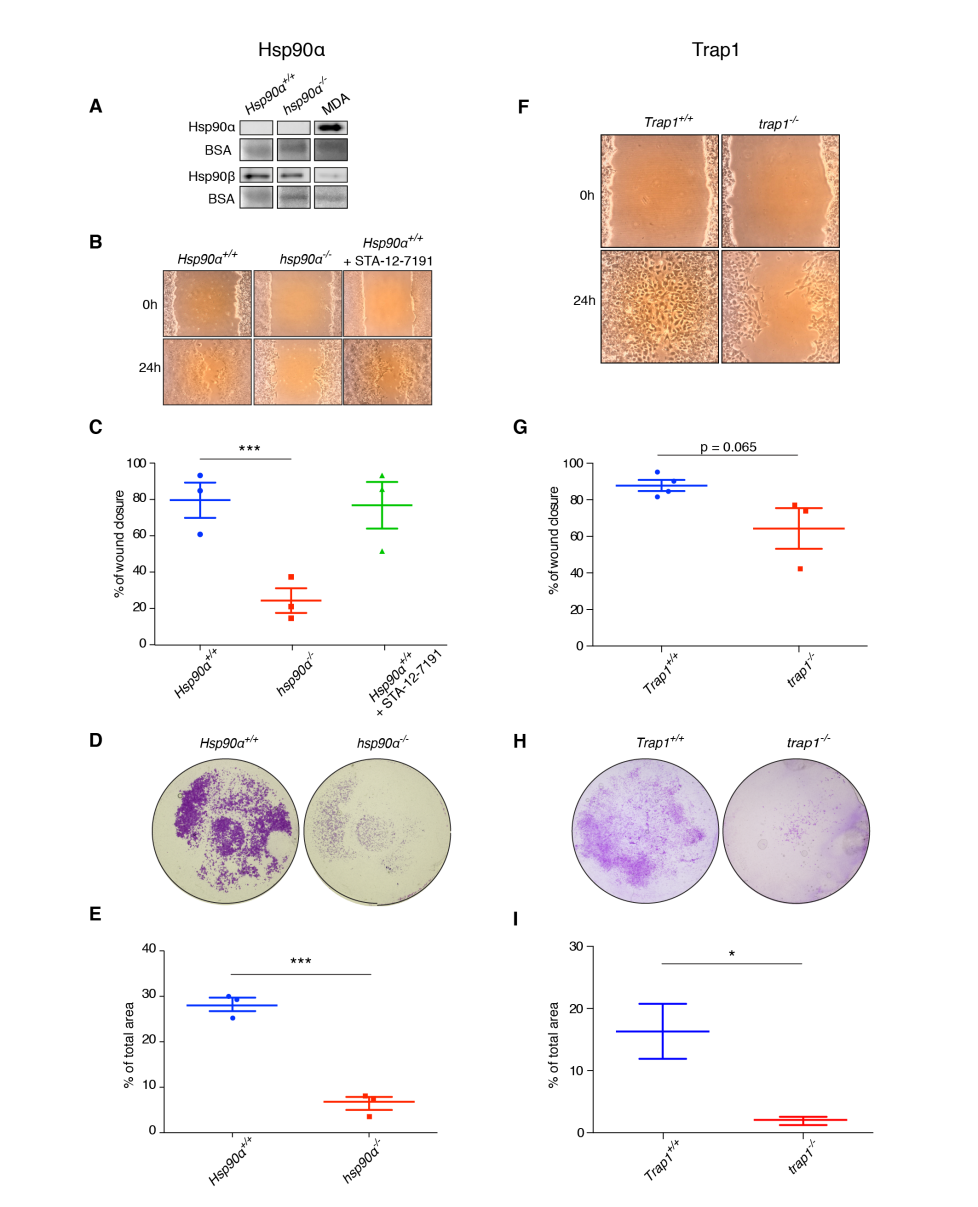
Mouse mammary tumor cells do not secrete Hsp90α, and lack of Hsp90α or Trap1 impairs migration and invasion **A**. Immunoblot showing the absence of Hsp90α, but the presence of some Hsp90β, in conditioned media from *Hsp90α*+/+ and *hsp90α*−/− cells; human MDA-MB-321 breast cancer cells served as positive control for Hsp90α secretion; BSA present in the medium served as loading control. **B**. and **C**. Scratch assay on plastic plates indicating significantly reduced migration ability of *hsp90α*−/− cells (***p < 0.001), but not of *Hsp90α*+/+ cells treated with the cell-impermeable Hsp90 inhibitor STA-12-7191 (100 nM). Micrographs **D**. and quantitation **E**. of transwell assays (***p < 0.001). **F**. and **G**. Scratch assay with *trap1*−/− cells showing a trend towards decreased migration (*p* = 0.065). **H**. and **I**. Transwell assay demonstrating that *trap1*−/− cells were significantly less invasive (*p < 0.05); two images per scratch at 0 and 24 hours were taken at 40x magnification with a Dino-lite camera (AnMo Electronics Corporation); the software ImageJ was used to measure the width of the scratches at five points per picture; for transwell assay photos were taken using a stereo microscope (15x magnification); Images resulting from duplicates of three independent experiments were quantified by ImageJ.

eHsp90, notably eHsp90α, has been suggested to play an important role for breast cancer cells to stimulate migration [12]. We therefore assessed the potential contribution of eHsp90 to the migratory behavior of our primary mouse carcinoma cells. Unexpectedly, we were unable to detect the presence of eHsp90α in conditioned media of *Hsp90α*+/+ and *hsp90α*−/− cells (Figure 5A). In contrast, eHsp90α from human MDA-MB-231 breast cancer cells was readily detectable, along with secretion of some Hsp90β from all three mouse cell lines. We further explored the possible involvement of eHsp90 pharmacologically by treating cells with the cell-impermeable Hsp90 inhibitor STA-12-7191 [38]. In the scratch assay shown in Figure 5B, both untreated wild-type (*Hsp90α*+/+) cells and cells treated with STA-12-7191 had a comparable capacity for migration. When MDA-MB-231 cells were used for a control experiment, the migration of these cells was significantly reduced by this inhibitor (Supplementary Figure 3), as previously described [38]. These experiments confirm the absence of functionally relevant eHsp90α, in particular, and eHsp90, in general, in *Hsp90α*+/+ mouse carcinoma cells.

### Compromised cell migration and invasion in mammary tumor cells without Hsp90α or Trap1

We wanted to check if the proliferation defect of *hsp90α*−/− and *trap1*−/− cells was accompanied by a change in migration and invasion of these cells. Therefore, we performed a scratch assay to compare the cells of different genotypes. Indeed, the absence of Hsp90α and Trap1 reduced the migration ability of mammary tumor cells, as both *hsp90α*−/− and *trap1*−/− cells were significantly less efficient in wound closing (Figure 5B, 5C and 5F, 5G, respectively). To study the invasive potential of the isolated cells, we used a transwell invasion assay. Compared to *Hsp90α*+/+ and *Trap1*+/+ cells, which were able to invade through the matrigel matrix into the lower chamber within 24 hours, both *hsp90α*−/− and *trap1*−/− cells displayed a significantly compromised invasive capacity (Figure 5D, 5E and 5H, 5I). These results indicate that the absence of Hsp90α or Trap1 impairs both migration and invasion of mouse mammary tumor cells.

### Correlation between higher levels of *Hsp90α* and bad prognosis in human breast cancer

**Figure 6:**
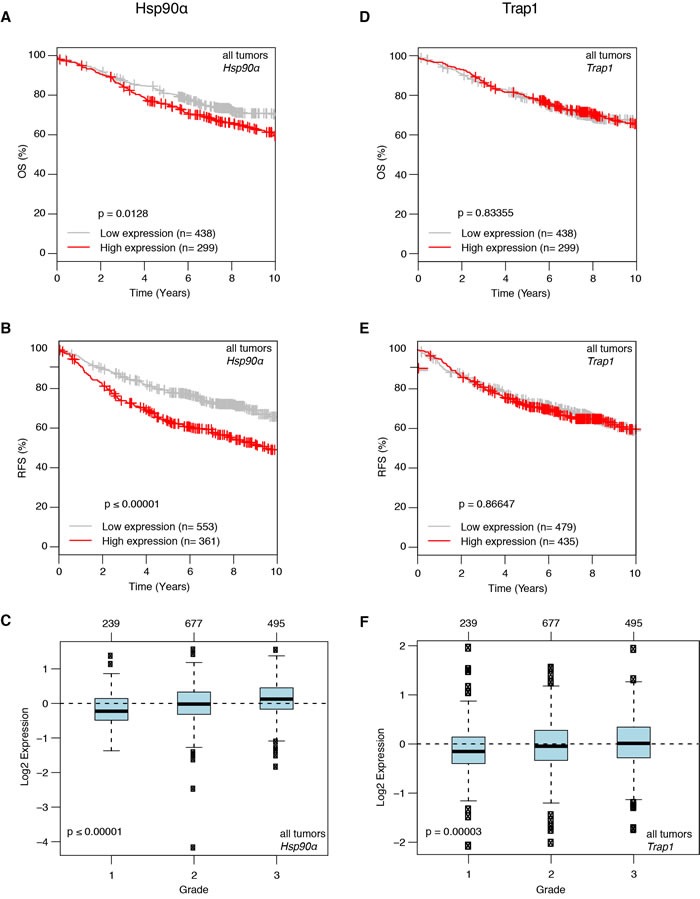
Correlation of higher ***Hsp90α*** mRNA expression with worse prognosis in human breast cancer revealed by a GOBO analysis. **A**. and **B**. Higher *Hsp90α* expression is associated with significant decrease in overall survival (A. *p* = 0.0128) and relapse free survival (B. *p* ≤ 0.00001). **C**. Higher *Hsp90α* expression correlates with increase in tumor grade (*p* < 0.00001). **D**. and **E**. *Trap1* mRNA expression and overall and relapse free survival show no correlation. **F**. Increase in *Trap1* expression is associated with higher tumor grade (*p* = 0.00003).

Having found that Hsp90α and Trap1 play a role in one particular mouse mammary cancer model, we wondered to what extent one could extrapolate to human breast cancer. As a first step towards this goal, we set out to investigate whether expression levels could be correlated with breast cancer features. For this purpose, we utilized the online tool “Gene Expression-Based Outcome for Breast Cancer” Online (GOBO) [39]. This tool allows one to interrogate a large and very diverse dataset of breast cancers for such correlations. One important caveat is that the underlying data are mRNA and not protein levels. With that in mind, the GOBO analysis revealed significantly reduced time of overall and relapse-free survival for patients with higher *Hsp90α* mRNA levels (Figure 6A and 6B), and therefore presumably Hsp90α protein levels, along with a correlation of significantly increased *Hsp90α* expression and higher tumor grade (Figure 6C). A similar analysis also revealed that increased *Trap1* expression is associated with higher tumor grade (Figure 6F), but failed to indicate a significant correlation with overall or relapse-free survival (Figure 6D and 6E). These findings are generally in agreement with the Hsp90α mouse experiments described above, and highlight the importance of Hsp90α for breast cancer development and metastasis in both mice and humans. In contrast, the results obtained for Trap1 require further investigations to clarify exactly which human breast cancer type may be represented by the mouse model with respect to Trap1.

## DISCUSSION

Hsp90 has been investigated for many years as a potential player involved in cancer pathogenesis; however, the exact details of its mode of action still remain elusive. Even less is known about the role of Trap1 in tumorigenesis. We aimed to address the issue of Hsp90α and Trap1 involvement specifically in breast cancer initiation, progression and metastasis by using an *in vivo* mouse breast cancer model. Such a mouse model provides more relevant information than previously described experiments with cancer cell lines and xenografts. We found that neither Hsp90α nor Trap1 are absolutely required for mammary tumorigenesis and metastasis, but have striking modulatory effects.

An important limitation of our approach that must be kept in mind is that the query proteins Hsp90α or Trap1 were absent both from the mammary epithelial cells, i.e. the tumor cells, and the stroma at the primary tumor site and all the way to the metastatic sites in the lung. Recent xenograft experiments have revealed a requirement for Hsp90α in the host for the colonization of the lung by Hsp90α-positive melanoma cells [40]. In the future, the challenge will be to dissect the functional importance of Hsp90α and Trap1 separately in the tumor cells, in the stroma and in the metastatic niche across a panel of tumor models.

### Could Trap1 and Hsp90α be redundant?

The fact that these molecular chaperones are not absolutely required in our mouse tumor model could formally be due to functional redundancy. While we cannot formally exclude this, there is no experimental evidence to support it. Trap1 and Hsp90α being only 29% identical, it is not very likely that they could replace each other, were they to be localized in each other's compartments. Trap1 is synthesized with an N-terminal mitochondrial targeting signal, but some “cytosolic” Hsp90 was reported to be present in mitochondria [27]. If this is also the case in our tumor model, then clearly it is insufficient to suppress all of the phenotype associated with the absence of Trap1. Moreover, the same authors argued that the effects of a Trap1 knock-down and of pharmacologically inhibiting mitochondrial Trap1 are the same [27]; one must conclude from these results that Trap1 and mitochondrially localized cytosolic Hsp90 are not functionally redundant, since the latter would also have been inhibited in mitochondria by the same inhibitor.

### Tumor initiation and growth

In the absence of Hsp90α, even though we did not observe any changes in the time of tumor initiation and age of mice at sacrifice, impaired tumor growth was apparent. Lack of Hsp90α made a difference for the dynamics of tumor growth, as *hsp90α*−/− mice showed a decrease in tumor burden, albeit not in tumor numbers. Further supporting this observation, Hsp90α-null cells isolated from primary mammary tumors had slower proliferation rates. These results can be related to previous reports on the inhibitory effects of Hsp90 inhibitors on breast cancer cell growth in mouse xenograft models [41–44] and in clinical trials [45, 46]. In addition, we also found that the protein levels of Hsp90α were significantly elevated in tumors compared to normal mammary glands, a finding compatible with our conclusion that Hsp90α contributes to breast cancer growth.

As far as the mitochondrial Hsp90 isoform Trap1 is concerned, despite its increased levels in tumors compared to normal mammary glands, tumors still formed even in its complete absence. However, we saw a delay in the tumor onset in *trap1*−/− mice, implying that Trap1 may play some facilitating role in tumor initiation. It is conceivable that *trap1*−/− tumor cells might be slow at boosting their initially potentially lower glycolysis [23] as part of their metabolic adaptation to the tumorigenic process. Eventually, at sacrifice, total tumor burden appeared similar between the two groups, indicating that beyond tumor initiation Trap1 is dispensable for subsequent tumor growth. We assume that the trend towards longer overall survival of *trap1*−/− mice may be due to the observed delay in tumor initiation. In apparent contradiction to what we observed with our genetic model, pharmacological inhibition of Trap1 and *TRAP1* deletion in a prostate cancer model resulted in inhibition of tumor growth [47, 48] and reduced tumor incidence [49], respectively. In previously reported xenograft assays performed with SAOS-2 osteosarcoma cells [50], MDA-MB-231 and MCF-7 breast cancer cells [29], and human esophageal cancer cells [51], knocking down Trap1 by RNA interference resulted in decreased tumor growth. Intriguingly, this mirrors the reduced proliferation rates, which we observed for mammary tumor cells isolated from *trap1*−/− mice. The apparent discrepancies further highlight the already known contextual role of Trap1 [21]. Further investigations will be necessary to dissect the contributions of Trap1 and its impact on cellular metabolism to the different stages of tumor growth, from the earliest stages of tumor initiation to the growth dynamics of tumor masses.

### Metastasis

In view of the proposed role of eHsp90α in promoting migration and invasion of cancer cells [7, 12, 14, 38, 52], we were particularly interested in determining the impact of Hsp90α ablation on the rate and extent of metastasis. We discovered that, while Hsp90α is not absolutely required for metastasis, mice lacking Hsp90α developed pulmonary metastatic nodules less frequently than their wild-type counterparts; *hsp90α*−/− mice also displayed lower numbers and smaller areas of metastatic nodules in the lungs. Furthermore, the migratory and invasive potential of cells isolated from primary mammary tumors was significantly decreased in cells lacking Hsp90α. These results point to an important role of Hsp90α for the metastatic progression and are in line with previous reports on Hsp90 acting as a potent enhancer of migration and invasion [7, 38, 52–59]. It has been speculated that some effects of Hsp90 inhibitors could be the result of the inhibition of eHsp90 rather than or in addition to the inhibition of intracellular Hsp90 [12, 17]. It is certainly reasonable to hypothesize that intracellular and extracellular pools of Hsp90 molecules perform distinct functions. Given that we could not detect appreciable amounts of eHsp90α with primary mammary tumor cells from wild-type mice, any Hsp90α function is most likely attributable to intracellular Hsp90α. It should also be emphasized that secretion of the other isoform, Hsp90β, which we could detect, obviously did not compensate either. The dependence on secreted Hsp90 and the signals such as stress and activated oncogenes that promote this secretion may vary from cancer to cancer [12, 17]. It is possible that the transformation by the PyMT oncogene in our particular model does not stimulate the secretion of Hsp90α. Hence, and although we cannot formally exclude that these same cells do secrete eHsp90α when they are within the context of a tumor in the mouse, we conclude that the observed growth, migratory and invasive defects of Hsp90α-null mammary tumor cells must be due to the lack of intracellular Hsp90α. Whatever the contribution of eHsp90 to human cancers may be, in our murine breast cancer model, it is the absence of intracellular Hsp90α, which compromises tumor progression and metastasis. The fact that metastatic tumors can still form in the absence of Hsp90α at all is intriguing. This may be due to compensatory mechanisms that are stochastically switched on in a small subset of cells. This might involve a permanent or temporary upregulation of expression or activity of the other Hsp90 isoform, Hsp90β, or of other cancer-relevant cellular processes.

For Trap1, differences between *in vivo* and *in vitro* results indicate a more complex situation. In the mouse, pulmonary metastases formed equally in both experimental groups, although there was a big intragroup variation. However, *trap1*−/− primary mammary tumor cells in culture showed a trend for slower migration and a strong defect in invasion. These differences are not related to the metastatic status of the mice from which the cells were isolated; as mentioned before, *Trap1*+/+ cells were obtained from animals that did not display the highest metastatic burden, whereas *trap1*−/− cells were derived from mice with very different metastatic loads. These results may indicate substantially different requirements *in vivo* versus *in vitro* regarding the cellular energy metabolism. In agreement with our results, Trap1 appears to promote cell migration and invasion in human esophageal squamous cell cancer [60]. However, these results contradict what we have previously found ourselves with *trap1*−/− mouse adult fibroblasts, and with HCT116 and HeLa cells depleted of Trap1 by RNA interference; these cells performed considerably better in a transwell assay [23]. Similarly, it has recently been published that invasive breast cancer cell lines have lower levels of Trap1; the same investigators found that when Trap1 is overexpressed in the invasive cell line MDA-MB-231 and cells are injected into the tail vein of immunodeficient mice, metastasis is inhibited [29]. Once again, as for primary tumor growth, context seems to be crucial for Trap1. The challenge for the future will be to carefully clarify the role of Trap1 in metastasis for different types of cancers both in tissue culture and in *in vivo* mouse models.

### Relevance to human breast cancer

A major difficulty in extrapolating from experimental data from a few cell lines to human cancer is their singularity. Using the online tool GOBO we found that an increase in *Hsp90α* mRNA levels correlated with bad prognosis in breast cancer patients. This is in agreement with previously reported findings for non-small cell lung cancer [61], cells from acute myeloid leukemia patients [62] and gastric cancer patients [63]. Contrary to what we observed in the mouse model, in patients there is no correlation between tumor-free or overall survival and *Trap1* mRNA levels. However, higher Trap1 expression correlated with higher tumor grade, which mirrors the functional relationship between Trap1 and tumor onset seen in our mouse model.

Overall, our findings provide further evidence for an important role of Hsp90α in breast cancer progression not only in our mouse model but also in human breast cancer patients. They corroborate the notion that Hsp90 should be considered as a target in anti-cancer treatment strategies. For Trap1, our results further emphasize the importance of the context and support the argument that targeting Trap1 may not be beneficial in all types of cancer at all stages and in all circumstances.

## MATERIALS AND METHODS

### Animals

Mice were housed under standard conditions, and all animal experiments were conducted in compliance with Swiss laws. The generation of the *hsp90α*−/− mutant mouse strain (*hsp90aa1*Gt(XE444)Byg) and the genotyping primers for this strain have been previously described [34]. *trap1*−/− mice, derived from the ES cell clone E041F05, were obtained from the German Gene Trap Consortium. The *Trap1* gene disruption is due to the insertion of a gene trap cassette (vector rFlipROSAßgeo +1) in the first intron of the gene (*trap1*Gt(E041F05)Wrst allele). Genotyping: PCR with forward primer 5’-gtcaagccctggggtaactacgg-3’ and reverse primer 5’-cactttaatcctaccactctgggg-3’ gives a band of 606 bp for the wild-type *Trap1* allele; PCR with forward primer 5’-aggggtctcccgatcccg-3’ and reverse primer 5’-cactttaatcctaccactctgggg-3’ gives a band of 538 bp for the mutated *trap1*Gt(E041F05)Wrst allele. FVB/N-Tg(MMTV-PyVT)634Mul/J (PyMT) transgenic mice [32] were a gift from Dr. Agnese Mariotti's laboratory (CHUV, University of Lausanne, Lausanne, Switzerland). *Hsp90α*+/−/PyMT males were crossed with *Hsp90α*+/− females to generate *Hsp90α*+/+/PyMT and *hsp90α*−/−/PyMT females used for the analysis. *Trap1/trap1*Gt(E041F05)Wrst*/PyMT* males were crossed with *Trap1/trap1*Gt(E041F05)Wrst females to generate *Trap1*+/+*/PyMT* and *trap1*−/−*/PyMT* females used for the analysis.

### Evaluation of tumors, tissue collection, histology and immunohistochemistry

From the age of 30 days, the development of mammary tumors in female mice carrying the PyMT transgene was followed by palpation for the appearance of the multifocal mammary adenocarcinomas thrice a week. When the tumors reached the legal size, mice were euthanized, number and size of tumors were determined, and tumors and lungs were collected and further processed. “Tumor number” represents the number of adenocarcinoma foci per mouse; “tumor burden” is defined as the total volume of the detectable tumor foci per mouse, a correlative measure of the number of cancer cells. Paraffin sections of tumors and lungs were subjected to H&E or immunohistochemical staining according to standard protocols using an anti-PyMT antibody (sc-53481; Santa Cruz Biotechnology).

### Histomorphometric analysis

For measurements of total areas of lung metastasis, four 5 μm thick sections separated by 100 μm were stained with H&E and photographed using a stereo microscope. The software ImageJ was used to calculate the total area and number of metastatic nodules normalized to the total lung area. Slides with adjacent sections stained with the PyMT antibody allowed for a confirmation of the presence of metastatic sites.

### Isolation of primary epithelial cells from mouse mammary tumors

Primary epithelial cells from mouse mammary tumors were isolated as previously described [64] and cultured in standard DMEM with 4.5 g/l glucose and 10% fetal bovine serum (FBS).

### Tissue and cell extracts and collection of conditioned media for immunoblotting

Proteins were extracted from frozen tissues according to standard protocols. To collect secreted proteins, *Hsp90α*+/+ and *hsp90α*−/− primary cell lines or MDA-MB-231 cells were seeded at the same density in OPTI-MEM medium (ThermoFisher Scientific) with 2% FBS; after 24 hours, cells were washed with PBS and grown for 48 hours in OPTI-MEM medium without FBS. Media were collected and centrifuged at 12 000 rpm for 20 minutes at 4°C, supernatant was concentrated using centrifugal filter units (10 kDa cutoff, Amicon Ultra) according to manufacturer's instructions.

Equal amounts of protein extracts or 40 μl of conditioned medium were subjected to SDS-PAGE followed by standard immunoblotting procedures. The following primary antibodies were used: anti-Hsp90α (EMD-17D7, Merck Millipore), anti-Trap1/Hsp75 (BD Biosciences), anti-Hsp90β (H9010, ThermoFisher Scientific), anti-Vimentin (PA5-27231; ThermoFisher Scientific), anti-E-cadherin (H-108, Santa Cruz Biotechnology), anti-PyMT antibody (sc-53481; Santa Cruz Biotechnology) and the pan-actin antibody C4 (Millipore). The secondary antibodies used were horseradish peroxidase-goat anti-rabbit or anti-mouse immunoglobulins (Dako).

### Proliferation assays

*Hsp90α*+/+ and *hsp90α*−/− cell proliferation was monitored by performing MTT assays with 3-(4,5-Dimethylthiazol-2-yl)-2,5-diphenyltetrazolium bromide (Applichem). The proliferation rate of *Trap1*+/+ and *trap1*−/− cells was determined by cell counting.

### Scratch assays

Cells were seeded at equal densities in standard medium, allowed to attach and treated for 2 hours with 5 μg/ml mitomycin C (Sigma). Afterwards, fresh standard medium without or with 100 nM of the cell-impermeable Hsp90 inhibitor STA-12-7191 [38] was added and three scratches per well were made in the cell monolayer with a sterile yellow tip.

### Transwell invasion assays

Cells were treated with 5 μg/ml mitomycin (Sigma) for 2 hours, trypsinised and seeded at an equal density in DMEM without FBS in the upper chamber of the transwell chamber (8 μm pore size) on 30 μl (Hsp90α cells) or 50 μl (Trap1 cells) of matrigel® matrix 1.5 μg/ml (Corning). The lower chamber was filled with standard medium. After 24 hours, cells were fixed in methanol and stained with 0.5% w/v crystal violet (Sigma) in methanol. The filter was cut out of the chamber and mounted on a slide.

### GOBO analysis

The online tool GOBO (Gene expression-based Outcome for Breast cancer Online) [39] was used to draw the correlation between expression of *HSP90AA1* (Hsp90α) (Entrez Gene ID: 3320) and *TRAP1* (Entrez Gene ID: 10131) and overall survival, relapse-free survival and tumor grade in microarray datasets of human breast cancer samples.

### Statistical analysis

Statistical analysis was performed with GraphPad Prism 6. Long-rank (Mantel-Cox) was used for the analysis of the Kaplan-Meier survival curves. The rest of the datasets were subjected to normality tests and compared by parametric (t-test) or non-parametric (Mann-Whitney) tests. When p-values were p < 0.05, differences were considered statistically significant. Error bars represent standard errors of the mean (SEM).

## SUPPLEMENTARY MATERIALS FIGURES


