# Supplementary material for: Cytosolic Hsp90α and its mitochondrial isoform Trap1 are differentially required in a breast cancer model
Source: Oncotarget. 2017 Feb 23;8(11):17428–42. doi: 10.18632/oncotarget.15659 (PMC5392260; doi:10.18632/oncotarget.15659)
Supplement: Supplementary file 1 [file oncotarget-08-17428-s001.pdf]

Cytosolic Hsp90α and its mitochondrial isoform Trap1 are differentially required in a breast cancer model

Supplementary Material

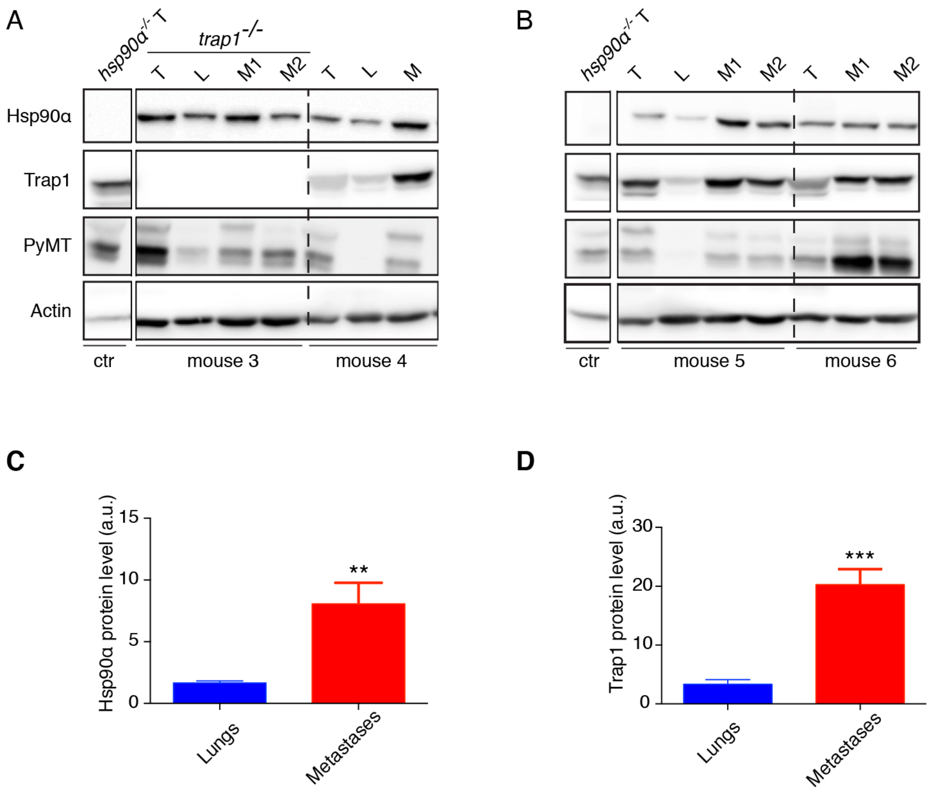

Supplementary Figure 1: A. and B. Immunoblots of Hsp90α and Trap1 in lungs (L), mammary tumors (T) and metastatic nodules (M). Companion data of Figure 1D. Extracts were derived from 4 mice; mouse 3 is trap1-/-; actin was used as loading control. C. and D. Quantitation of the immunoblots; significant increase of Hsp90α (** $p<0.01$, n=6 mice) and Trap1 (*** $p<0.001$, n=5 mice) protein levels in metastatic nodules compared to normal lung.

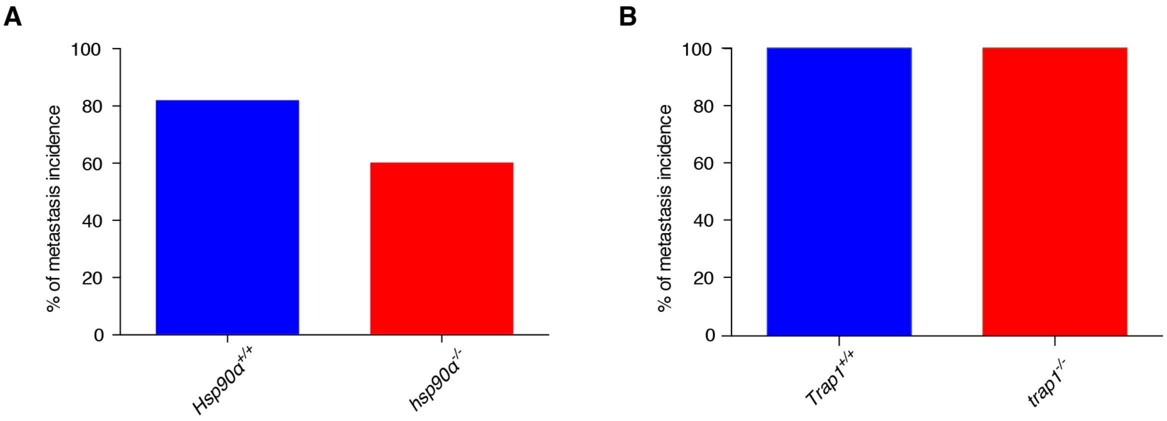

Supplementary Figure 2: Metastatic incidence in lungs. A. Comparison of 11 Hsp90α+/+ and 10 hsp90α-/- mice. B. Comparison of 7 Trap1+/+ and 7 trap1-/- mice.

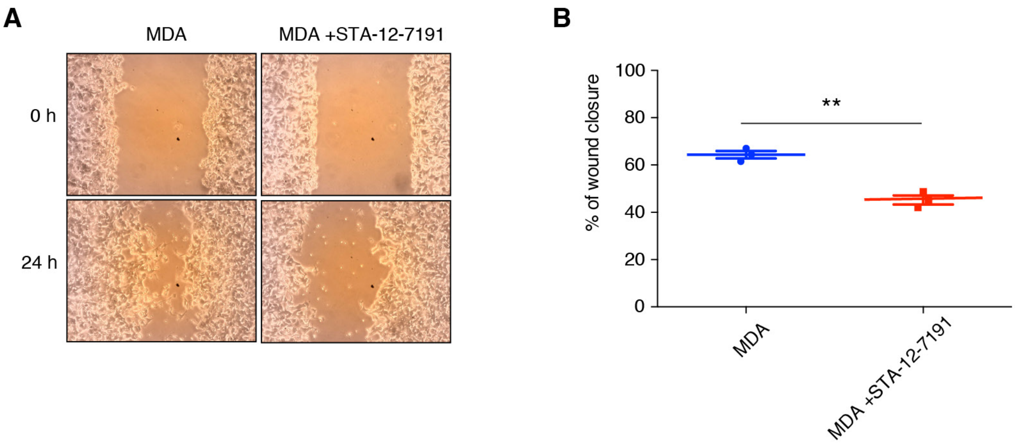

Supplementary Figure 3: Decreased migration of human MDA-MB321 breast cancer cells treated with STA-12-7191. A. and B. MDA-MB321 cells treated with 100 nM STA-12-7191 migrated significantly less than untreated cells (** $p<0.01$); images were taken at 40x magnification and are representative of three independent experiments.
